# Beyond the pilot phase: exploring the sustainable implementation of artificial intelligence in the English NHS

**DOI:** 10.3389/fdgth.2026.1743376

**Published:** 2026-02-19

**Authors:** Nilangi Patil, Charitini Stavropoulou

**Affiliations:** 1 School of Health and Medical Sciences, City St George’s, University of London, London, United Kingdom; 2 Centre for Health and Care Innovation Research, City St George’s, University of London, London, United Kingdom

**Keywords:** artificial intelligence (AI), innovation, NHS, pilot studies, sustainable implementation, public funding

## Abstract

**Background:**

We explore the experiences of Artificial Intelligence (AI) innovators who had received funding to pilot their innovation in the English NHS, with the aim of understanding what hinders and supports, from their perspective, the sustainable implementation of their innovation beyond the funding period.

**Methods:**

We first identified a list of companies that had received funding from two national schemes supporting AI innovations in the NHS, focusing on early rounds of these schemes. We then used personal contacts to identify key individuals from these companies, and used a snowball approach as well as LinkedIn contacts to increase our sample. We interviewed participants individually, using semi-structured interviews and analysed the data thematically.

**Results:**

We interviewed 18 individuals from 11 AI companies, who had received funding from two national schemes. Our findings show that the funding offered the companies a unique opportunity to pilot their innovations, show early successes and grow recognition around AI and its potential. Yet, innovators faced several barriers in their effort to implement their AI innovations beyond the pilot phase, including misaligned expectations regarding the programmes’ goal, fragmented adoption efforts with little national coordination, and inadequate evaluation mechanisms to generate the evidence needed for wider adoption.

**Conclusion:**

The UK has set great ambitions for the adoption of AI in the NHS and has invested significantly in public funding to support its use. Our findings show that public investment alone is not sufficient to achieve this ambitious target. A better understanding of the implementation challenges of using AI innovation in practice is needed.

## Background

In a rapidly evolving landscape, artificial intelligence (AI) is changing the way we perceive innovation in healthcare, offering transformative opportunities to enhance patient care, optimise clinical workflows, and drive operational efficiency (1, 2). Significant advancements in AI-driven technologies are gaining traction across diverse healthcare domains (3). In diagnostic imaging for example, AI-powered algorithms are showing promising results in detecting anomalies, such as tumours and fractures, while significant gains can be offered when AI takes over administrative tasks (4, 5).

As a result of the growing interest for these technologies, investment in AI in healthcare is expanding. Venture capital funding and private investments in AI-driven healthcare start-ups have soared in recent years, underscoring the growing confidence in the transformative potential of these innovations (6). At the same time, governments and healthcare organisations have also invested significant in AI solutions (7). Public funding has always been instrumental in advancing and integrating technology into healthcare systems and AI is no exception (8).

Following an international trend in investing in AI innovations, the UK government has vowed to be a global leader in “fairly and effectively seizing the opportunities of AI” (9). Public investment in this area has grown, with various councils, national funders as well as charities supporting AI innovations across a number of areas of healthcare (10), and more funding being invested (11). Yet, much of this funding focuses on the technical capabilities of AI and its clinical outcomes, with limited funding offered to understand how these technologies transition from concept to sustained implementation over time.

Meanwhile, many AI solutions struggle to gain traction, leading to slower adoption despite the technology's potential (12). There are growing concerns that AI implementation often encounters compatibility issues with existing systems and IT infrastructures (13), organisational resistance to change, and the requirement for a solid evidence base (14). Issues of trust and ethical considerations (15, 16) as well as limited access to unbiased data (17) have also been identified as key challenges when it comes to the adoption of AI in healthcare.

While the literature in this area is expanding, a systematic review of stakeholders' perspectives on the implementation of clinical AI highlights an “underrepresentation of perspectives from stakeholders other than healthcare professionals (HCPs)” and calls for greater empirical evidence from innovators. Notably, innovators' perspectives were examined in only 7.7% of the studies reviewed (18). In the absence of a clear understanding of implementation challenges that includes the innovators' standpoint, investment in AI research is unlikely to reach its full potential, contributing instead to “pilotitis”, the unnecessary repetition of pilot studies that do not lead to the wider spread of innovation (19).

## Methods

### Aim

This study aims to explore the experiences of innovators on what supports or hinders the sustainable implementation of AI-based innovations beyond the pilot phase in the English NHS. By focusing on the innovators' experiences, we contribute to a limited literature that shows how innovators who have received funding often encounter unique hurdles when transitioning from initial development to widespread adoption (20).

### Research design

This study adopts a qualitative approach, using semi-structured interviews to investigate the experiences innovators who work in AI start-ups face in sustaining their innovation within NHS. The interview schedule is available as a Supplementary File S1. A qualitative approach is particularly well-suited for this study, as it allows exploration of how AI innovators comprehend and construct their experiences within the NHS ecosystem (21).

### Sampling and recruitment

Recruitment happened in two stages. First, using publicly available information, we identified AI companies that had received funding from two national schemes supporting AI innovation in health and care settings. Both schemes aim to accelerate the adoption and integration of AI within the NHS by offering structured financial support to innovative enterprises. Funding is provided in phases, starting with feasibility studies, progressing to larger scale testing, evaluation and implementation support. We initially focused on projects that were in later stages of their innovation, representing AI technologies in the medium stage that have market authorisation but insufficient evidence to deploy on large scale. Later, some early-stage projects from one of the schemes, were also included. This offered us the opportunity to explore whether some of the issues identified from initial interviews, were also observed in earlier stages of the innovation journey. Financial support was given to these projects to run pilot studies to provide evidence that would ultimately support their national roll-out. We identified companies from early rounds of these schemes, as our interest lied in exploring not only the implementation challenges, they faced during the award, but also their innovation journey after the funding was over.

Second, having identified a list of companies, we used personal contacts to facilitate introductions to project leads and key individuals from these companies. We expanded on the participant pool using a snowball sampling method (22) and we also directly contacting individuals via LinkedIn. We included individuals who were directly engaged with the development, management, or implementation of the AI technologies during the funding period and who were willing to participate in the study. Participants who were not part of these projects or did not meet the inclusion criteria were excluded. Participants were sent an information leaflet informing them of the purpose of the study and were asked to sign a consent form prior to the interview.

The study was part of one of the author's MSc dissertation project (NP) and was supervised by CS. The study received ethical approval from City, University of London - the Health Services Research & Management Proportionate Review Committee (ETH2425-0632).

### Data collection

A semi-structured interview guide was developed with open-ended questions and prompts aligned with the research objectives. The interviews were conducted one-to-one by one of the authors (NP), primarily via Microsoft Teams based on the participants' preferences and availability and were audio-recorded. The interview guide was adjusted after the initial two interviews to further refine the questions and prompts based on emerging themes and insights.

### Data analysis

Thematic analysis was chosen for the analysis for its capacity to uncover patterns and themes within the data, which allows for an in-dept exploration (23). Initial data familiarisation began immediately after each interview by one of the authors (NP), during the editing of the transcripts. Key parts of the interviews were identified and highlighted, then compiled into a separate document to guide initial coding process.

Guided by an inductive approach, NP undertook a first round of open coding (24), where simple codes were generated for all the interviews using quotes from the transcripts. This stage involved breaking down the data into meaningful segments to reflect participants' experiences. These open codes were systematically recorded in an excel sheet for transparency and organization. Following this the researcher aggregated these codes into first-order codes by identifying common patterns in the data. NL and CS discussed the codes and agreed on the final coding.

The emerging patterns were then reviewed and synthesised into broader sub-themes, resulting in a coherent representation of the data. These sub-themes were further refined and abstracted into four key themes, capturing the highest level of abstraction in the data structure. This process involved continuously revisiting the data, challenging the emerging categories, and assessing the fit of each data fragment into existing constructs.

### Patient and public involvement statement

Patients or the public were not involved in the design, or conduct, or reporting, or dissemination plans of our research.

## Results

A total of 18 interviews with innovators from 11 different companies funded by two national schemes were conducted between July 2024 and February 2025. Each interview lasted approximately 40 min. Table 1 presents the job titles of the participants, highlighting a variety of backgrounds and areas of experiences.

**Table 1:** Participants interviewed and their job descriptions.

National scheme	Company	Innovator	Innovator's job title
A	C1	I1	Business development manager
		I6	Business development manager
	C2	I2	Senior product manager
		I5	Medical director
		I7	Senior partnerships manager
	C3	I3	Data product manager
		I4	Senior machine learning engineer
	C4	I8	Director of operations
		I9	Program manager
		I10	Head of marketing
	C5	I15	Chief operating officer
		I17	PMO manager
	C6	I16	Clinical program manager
B	C7	I11	CEO
	C8	I12	Senior client partner
	C9	I13	Co-funder, business and finance leader
	C10	I14	Funder and CTO, previously CEO
	C11	I18	Director of Clinical and Commercial Development

Companies participating in Scheme A received funding in 2020, whereas those in Scheme B were funded more recently, between 2021 and 2023. At the time of the interviews, all projects funded under Scheme A had been completed, while projects in Scheme B were either ongoing or in the process of applying for additional funding.

The companies represented a wide range of innovation types and clinical specialities. Two companies specialised in cardiology and two in radiology; one each focused on nephrology, pathology, neurorehabilitation, and respiratory medicine, while three primarily supported the operational aspects of healthcare delivery. Five innovations were diagnostic tools, three targeted workflow optimisation or administrative processes, two supported clinical decision-making, and one was a monitoring device.

Companies in scheme A, were funded with the clear aim to provide the evidence that would support national roll-out. Our analysis revealed that none of these innovations had plans for national roll-out beyond the pilot phase. Two companies (C2, C5) discontinued their operations in the UK after the pilot, and focus their efforts on other international markets.

“…we just stopped. I mean, so again, this is UK. So, we focused on the US where you know the financing is better, the willingness to pay us higher. So, we just simply shut it down and moved on. So, we're doing, we have no engagement on the NHS after that project.” (I15, Chief Operating Officer, C5)

Of the remaining three companies (C1, C3, C4) none had yet secured further funding or established a clear strategy for continued NHS adoption, though discussions were reported to be underway.

“So I'd say we're hanging in there, but we're hanging in there for, essentially, national adoption. I think if in six, nine months’ time, there isn't some movement on that, we're in real trouble.” (I8, Director of operations, C4)

From scheme B, three early-phase companies (C9, C10, C11) had completed their current funding stage and had already applied for the next phase, awaiting its commencement. Although this phased funding was designed to support innovation and growth, the start-ups faced challenges in securing sustained investment impacting their ability to scale their innovations with the NHS.

### Key findings

**Figure 1:**
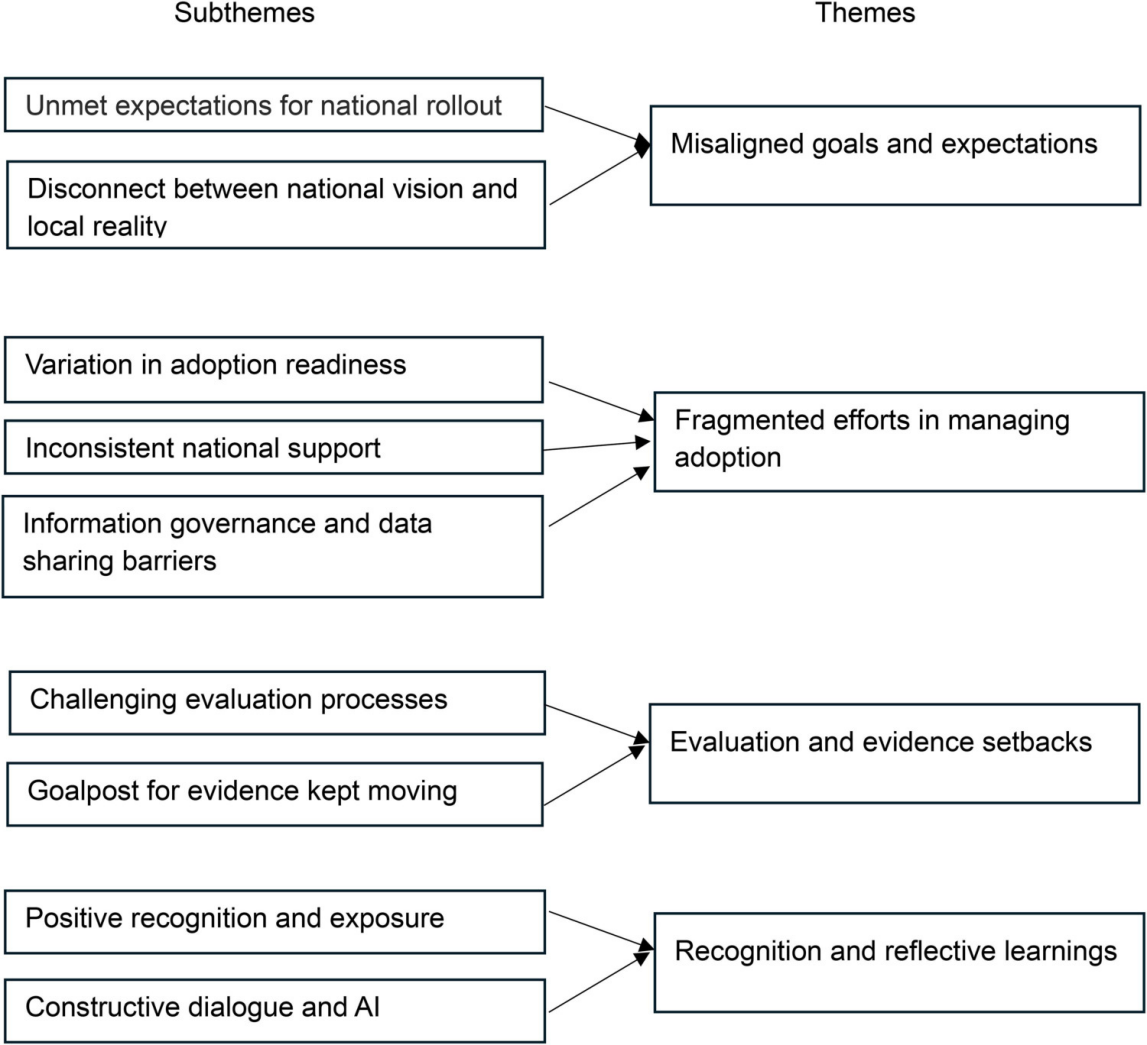
Main themes and subthemes.

The interviews highlighted several key insights into the process of implementing a new AI intervention within a healthcare system. Participants shared their experiences and perceptions revealing challenges encountered during the innovation's rollout. These narratives provided a rich understanding of how the innovation was perceived, the level of engagement it received, and the practicalities of integrating it into existing workflows (Figure 1).

#### Misaligned goals and expectations

##### Unmet expectations for national rollout

The programmes' ultimately goal was to allow companies to generate the necessary evidence to then allow the national roll-out of the innovation. Yet, participants felt that there was little that was done to support this in practice.

“At the beginning of the project, we were informed that there would be a path towards a local tariff or a mechanism essentially that would allow us to then, if it was successful, to spread the net and allow adoption on a broader scale. That has not been forthcoming.” (I6, Business development manager, C1)

“…that's a bit of a disconnect between the stated aims of the award, which implied that by the time we got to the award, if you were successful, then you would be moving forwards on a pathway to commissioning.” (I5, medical director, C2)

This created a sense of stagnation and innovators were left unsure about the practical steps required to bring the AI tools to market, including reimbursement models and commissioning pathways. This inconsistency in expectations made it difficult for the innovators to progress towards commercialisation, leaving many uncertain about the future of their projects. Despite discussions about creating a reimbursement mechanism, there was a lack of concrete action from the NHS, as another participant noted,

“There needs to be a much clearer path from the AI team, from the [national body] in terms of how they intend to create a reimbursement mechanism. OK, they talked a lot about that, but it never transpired.” (6, Business Development manager, C1)

Yet, as the participants highlighted this is not only about reimbursement, but also around the lack of coordinating efforts at a national level to influence policy and prepare the ground for an adoption pathway:

“The funders were very supportive but they weren't able to drive a conversation with the [policy maker], and none of [funders] there were in a position to motivate the [policy maker] to directly inform the process. They'd give out general guidance about what they're looking for from all of AI evidence, but there's no one in that system who's driving AI adoption other than the funders who gave some money and don't have a lot of power to do anything else.” (I5, Medical Director, C2)

##### Disconnect between national vision and local reality

The great expectations underpinning these schemes that these innovations would move quickly into NHS were hit by realistic obstacles in practice. Innovators talked about a “*misalignment between the intentions of the [award] and the understanding at local level what that then means.” (I2,* *Senior product manager, C2).* This led to practical challenges. Contracting issues contributed to significant delays, as getting agreements in place often proved to lengthy and complex process. These contractual deliverables were set at a high level, but the execution on the ground required much more detailed work to align with the operational realities of each site.

“It took us nearly a year to get the first site to contract. This was because of the mixed goals between what the [funder] wanted out of the [award] and what sites were willing to sign up for.” (I7, Senior partnerships manager, C2)

#### Fragmented efforts in managing adoption

##### Variation in adoption readiness across sites

Each site approached the AI intervention with its own unique set of priorities and challenges, making implementation a slow and laborious process. This was not helped by the fact that the majority of the companies we interviewed received their funding just before the COVID-19 pandemic and had to slow the implementation processes due to the restriction imposed during that period. Still, variations across different sites created additional hurdles in moving the intervention forward and there was considerable pushback from sites when they realised the costs they would incur once they had to fund the service independently.

“Every site has a different appetite for these kinds of projects. Every site has different resource constraints to support these kinds of projects. Every site has a different understanding of AI and of information governance and of the technical requirements. So, it's been completely different everywhere we have been.” (I7, Senior partnerships manager, C2)

As one participant explained, very often sites wanted to be involved, because they were interested in understanding the technology and be part of the research around it, but they were not necessarily ready to adopt it.

“We won [an award], which was technology that was ready to go, put in deployed to then move into phase five which is commercial adoption, none of the clinical sites themselves are on that journey, or on the same stage of that journey. They wanted to be involved in research. They wanted to understand how the tool works. They want to get a feel for it. None of them are ready, or have the ability to actually buy it because of commissioning rules, because of procurement processes, and because there isn't a clear recommendation from a national body to then say this technology can be purchased.” (I2, Senior Product Manager, C2)

##### Information governance and data sharing barriers

A recurring theme in the interviews was the struggle to navigate critical issues such as Information Governance (IG) and data sharing, all of which significantly hindered the momentum required for successful long-term engagement. Initial commitment, particularly as the AI tools were framed as a means to enhance efficiency and patient care, was followed by significantly delays by data-sharing and governance issues. One participant noted,

“There is often clinical enthusiasm, but the actual trusts were not comfortable to make the step forward into getting engaged for data sharing particularly.” (I5, Medical director, C2)

Data management itself emerged as a significant obstacle with participants citing difficulties in obtaining high-quality data across NHS sites. Pointing to the inconsistency in data standards across different NHS providers as a barrier to effective implementation as many AI projects depend on robust data access to refine their models and demonstrate value.

“…what we found that is with each of these five different trusts, there were five different teams handling this information governance process” (I12, Senior client partner, C8)

Additionally, concerns were raised about limited visibility into data-sharing processes, as another participant noted, “We were getting out three times as much data as we were supposed to get…they actually have very poor sight of what they were sharing.” (I*5, Medical director, C2).*

Collecting the benchmarking data, which was essential for tracking AI performance lacked sufficient support leaving teams without the necessary data to measure progress. Many NHS sites operated with old IT systems, or relied on paper-based processes, creating a disjointed environment that made technology integration difficult. The legacy IT systems required significant intervention, which led to delays in the implementation process. Although there was interest in adopting AI innovations, many sites were unable to proceed due to a lack of sufficient resources. As one participant noted, “*a lot of the sites that couldn't get involved couldn't do so purely because of resource constraints.”* (*I7, Senior partnerships manager, C2*).

##### Inconsistent national support

Participants talked about the lack of national coordination efforts to support the implementation process. For example, efforts to secure information governance support from the NHS were largely unsuccessful, leaving innovators to navigate these complex issues without adequate guidance. This forced the innovators to rely on external resources to navigate governance hurdles, ultimately contributing to further delays and financial strain.

Instead, when there was national support, things moved on more smoothly. One company struggled to engage with the different sites until the national coordinating team got involved in the process to support them.

“There was a realization about halfway through the project where [national funder] said, ‘we’re going to be really held to account if we're not properly supporting when we are giving them an award of strategy millions.” (I5, Medical director, C2)

This shift in approach led to a more streamlined onboarding process, reducing the time required to onboard sites from several months to just a few weeks, ultimately facilitating smoother adaptation.

#### Evaluation and evidence setbacks

##### Challenging evaluation processes

All projects were subject to external evaluation. The evidence was crucial to support future recommendations for the AI innovations to national bodies like NICE. A consistent concern raised by most companies was the challenges faced with the evaluation process. Frequent personnel changes and a lack of understanding of the AI-technology further complicated the process, leading participants to feel concerned that the final assessment might be flawed. The differing expectations between the evaluation team and the AI company's agile approach created additional challenges, as some decisions made were viewed to be potentially impacting the final evaluation.

“We were meant to collect data from both a [disease A] cohort of patients and a [disease B] cohort. In the end, [the evaluation team] only put data from the [disease A] cohort, so all the data that we collected was, a lot of it of dismissed, either because they didn't like the data, or it wasn't coherent enough across different trusts. Some of them had different pathways so it was a bit difficult because there were a lot of comparatives that were different. They still haven't published their final report, despite it being over a year since completion, so it hasn't felt particularly collaborative.” (P6, Business development manager, C1)

There were significant delays with the evaluations and as one participant noted “five years later there is no NICE evaluation” *(I8, Director of operations, C4).*

Issues with evidence generation started early on in the journey, even for companies in phase 2 or 3, who were trying to navigate the innovation journey:

“We also worked with [name of company], which was a health economics company. And they helped us complete the meta tool. To be quite honest with you, that was not useful at all .. we understood a few things from that, but the partners that we had there were not useful. I mean, they were like speaking a lot of jargon and didn't quite put themselves in startups shoes.” (I13, Co-funder, business and finance leader, C9)

##### Goalpost for evidence generation kept moving

One of the main aims of the programmes was to support the companies generate the evidence needed for the NHS to make decisions on whether their innovation could be rolled out nationally. Yet, innovators highlighted how the expectations for the evidence required kept changing and “*the goalposts for the evidence requirements from the committees that make these decisions has moved throughout the period of time that we've been doing this programme of work” (I1, business manager, C1).* One participant highlighted how these changes were requested at the end of the project, which meant that a new approach had to be adopted to generate the evidence the funder wanted.

“…the expectation from the outset was if we had done all that, and delivered everything, [the company] would be able to commercialise our product. We would then be able to sell it to the NHS for it then to be used. That is not the case. We have delivered what we said we would do, and we've done what we said we would do, […] They are still wanting more evidence. What we did was we worked with them and other key stakeholders and said, ‘Well, what evidence is it you'd want to see beyond what we've already produced?’ We designed a protocol for a new study called XXX which is a randomised controlled trial, because they said, ‘What we want to see is a randomised controlled trial.”‘ (I2, Senior product manager, C2)

#### Recognition and reflective learnings

##### Positive recognition and exposure

All the participants highlighted the recognition that the AI award brought to their projects, which significantly boosted their visibility and credibility. The award not only provided financial support but also helped them with networking and exposure, with one participant noting that the initiative “*generated a lot of good publicity, including national news and society exposure*.” (*I6, Business development manager, C1)*

It also helped them improve their project. User feedback has played a crucial role in that respect in shaping the evolution of the AI products. One participant noted, “*There were a lot of reiterations done, optimizations based on the feedback we had received”* (*I4, Senior Machine learning engineer, C3).* These learnings helped them address specific challenges and improve the overall functionality of the product. As they noted, numerous user interviews were conducted, helping the team clear up assumptions and continuously improve the AI tools based on feedback.

##### Constructive dialogue and AI acceptance

Despite the challenges faced, the growing acceptability of AI technology within NHS was seen as a positive outcome. One participant described the shift in attitudes: “*there's been a massive shift in acceptability of AI. Even during the three years of the award, the people are they start to understand how it works…which they didn't before*.” (*I5, Medical director, C2).* The successful deployment of AI technology at multiple sites played a pivotal role in fostering constructive dialogue and increasing acceptance among stakeholders.

Local champions were instrumental in that respect, helped companies navigate the difficulties of implementation and advocated for the adoption of their innovation:

“I think that bit's gone quite well because at each site what really helps is if you have those champions who want to understand question and help their colleagues adopt to the use of the AI” (I12, Senior client partner, C8)

## Discussion

The AI Opportunities Action Plan presented to the UK Parliament in January 2025 sets great ambitions for the adoption of AI “to boost economic growth, provide jobs for the future and improve people's everyday lives” (9). The UK has invested significantly in public funding to support the use of AI in healthcare and has vowed to be a global leader in AI innovation.

Our findings show that public investment alone is not sufficient to achieve this ambitious target unless a coherent strategy is built around the implementation of these innovations in practice. Much of this funding is provided for the upstream phase, which is crucial to increase the variety and capacity of the innovation ecosystem, but without a clear pathway to wider adoption, much of this funding ultimately will fail to land successfully as sustainably implemented changes in services or patient benefits.

The companies that we analysed in this study, faced significant challenges during the implementation phase, including misaligned expectations, fragmented efforts for adoption and evidence challenges and no company had clear plans for national roll-out beyond the pilot phase. Many of these challenges are not unique to AI innovations. There is plenty of evidence suggesting that innovation processes in the public sector are very complex and there is often a misalignment between the intended outcomes described a strategic level and on-the-ground challenges (25) similar to what our participants experienced. Adoption processes are also complex, and direct engagement with different adoption sites is crucial in the early stages of innovation implementation, as it helps to align stakeholder expectations with the goals of the innovation, creating a shared understanding and commitment (26).

Perhaps more specific to AI are issues around Information Governance and data sharing. The AI innovations are heavily reliant on data-driven algorithms, and the availability of high-quality data is crucial (27), highlighting the need to ensure the NHS is digitally mature to adopt these technologies in practice. There are concerns the NHS is not there yet (28) and that NHS providers struggle to move from “analogue to digital” due to inadequate IT infrastructure, limited budgets and day-to-day pressures (29).

Our findings are in line with recent studies that suggest a more cautious approach to the adoption and implementation of AI innovations is needed. A recent study looking at the state of adoption of AI across NHS primary and secondary care provider organisation in London, showed that adoption is still very slow and it is happening in an *ad hoc* manner and call for a national AI strategy in healthcare (30).

Without collective, joined-up efforts, AI technologies are unlikely to offer the desired outcomes. This highlights the need for unified leadership and strategic alignment within the NHS to ensure that AI tools are not only implemented but also thrive over time, delivering consistent value across different healthcare settings (31–34). Such initiatives should consider the funding and reimbursement mechanisms that are needed to be in place if the evidence suggests that the innovation offers value for money (6, 35). Effective evaluation mechanisms that can ensure accurate data collection, meaningful analysis, and actionable insights are needed to support the ongoing development and integration of AI technologies (32).

The NHS is under immense pressure from rising patient demand and significant workforce shortages and AI, though not a panacea, can help with some of these challenges (36). Without a comprehensive strategy these investments can result in fragmented efforts and hindered process (31). As Lord Darzi highlighted in his report, “there will need to be a fundamental tilt towards technology” for the NHS to fully capture the transformative potential of AI (37). This shift is essential for ensuring AI innovations continue to evolve and bring meaningful improvements to clinical pathways, patient care, and overall healthcare outcomes.

### Limitations

This study is not without limitations. The sample size is relatively small; however, the number of participating companies, the diversity of innovation types, and the range of clinical specialities in which they were applied allowed for a degree of heterogeneity. Another limitation is that projects funded under Scheme B were ongoing at the time of data collection, and further research is needed to assess the longer-term outcomes of these innovations once funding has ended.

## Conclusions

AI innovations in healthcare are often presented as the panacea for most medical challenges faced by health services. The UK has vowed to become “world-leader” in AI innovation and has invested significant in such technologies. Against this hype, our study suggests that significant financial investments into the technology are not enough to achieve sustainable implementation over time. More coordinated efforts among stakeholders are needed to bring promising AI technologies into the NHS.

## Data Availability

The datasets presented in this article are not readily available because of commercial confidentiality. Requests to access the datasets should be directed to the corresponding author.

## Author's note

This report is independent research supported by the National Institute for Health and Care Research ARC North Thames. The views expressed in this publication are those of the author(s) and not necessarily those of the National Institute for Health and Care Research or the Department of Health and Social Care.
