# Supplementary material for: Beyond the pilot phase: exploring the sustainable implementation of artificial intelligence in the English NHS
Source: Front Digit Health. 2026 Feb 19;8:1743376. doi: 10.3389/fdgth.2026.1743376 (PMC12964262; doi:10.3389/fdgth.2026.1743376)
Supplement: Supplementary file 1 [file Supplementaryfile1.docx]

**Interview Guide**

**Background information**

1. Could you please tell me about your role and your involvement with [company name]?
   1. Prompt; if not in the company any longer, what was their role when the project was funded and implemented.
2. Could you please tell me about the company and [product name]?

**Pilot phase**

1. Your company received funding from [funding body name] to implement and pilot [name of product]. Could you tell me more about the pilot?
   1. Prompt: What were the primary objectives?
   2. Prompt: What kind of support (technical, advisory, etc.) did you receive from the NHS during the project?
   3. Prompt: What were the key milestones and timelines?
2. Were there any specific challenges or obstacles you encountered during the project implementation? How did you overcome them?
3. How did you engage with key stakeholders within the NHS during the project? What were their main concerns or priorities?
   1. Prompt: How did you communicate the purpose and benefits of the AI tool to NHS staff and stakeholders?

6. Can you describe your experiences with the independent evaluation of your technology, including any challenges, outcomes, or impacts it had on your development and scaling processes?

**Post-implementation phase**

1. How has the project progressed after the initial funding ended?
   1. What have been the major developments achieved post-implementation?
   2. Have there been any new partnerships, collaborations, or business opportunities that emerged after the implementation phase?
2. Have there been any significant changes or pivots in your project's direction or focus since the implementation phase?
   1. Prompt: did the project continue to receive support or funding from other sources after the NHS program concluded?
3. Did you encounter any unforeseen challenges or obstacles after the implementation phase? How were they addressed, and how has the project evolved or adapted since the initial implementation phase?
4. How has the adoption of the AI tool been among NHS staff post-implementation?
   1. Prompt: What kind of training and resources were provided to NHS staff to facilitate the use of the AI tool?
   2. Prompt: How was the integration of the AI tool into existing NHS systems managed?

**Future plans and lessons learned**

1. Are there plans to scale up or expand the project beyond its current scope in the post-implementation phase?
2. Reflecting on your overall experience, how much has the pilot phase helped the adoption of the [product name] in the NHS?
   1. Prompt: If you could go back and make changes to the project post-implementation, what would you do differently?
3. Is there any additional insight or experience you would like to share regarding your project post-implementation, and what advice would you offer to other AI start-ups navigating the similar paths?
